# Direct Measurement of Diffusion in Olfactory Cilia Using a Modified FRAP Approach

**DOI:** 10.1371/journal.pone.0039628

**Published:** 2012-07-10

**Authors:** Mihai Alevra, Peter Schwartz, Detlev Schild

**Affiliations:** 1 Department of Neurophysiology and Cellular Biophysics, University of Göttingen, Göttingen, Germany; 2 German Research Foundation Research Center for Molecular Physiology of the Brain, Göttingen, Germany; 3 German Research Foundation Cluster of Excellence 171, Göttingen, Germany; 4 Department of Anatomy and Embryology, University of Göttingen, Göttingen, Germany; German Cancer Research Center, Germany

## Abstract

The diffusion coefficient of fluorescein in detached cilia of *Xenopus laevis* olfactory receptor neurons was measured using spatially-resolved FRAP, where the dye along half of the ciliary length was photobleached and its spatiotemporal fluorescence redistribution recorded. Fitting a one-dimensional numerical simulation of diffusion and photobleaching for 35 cilia resulted in a mean value of the diffusion coefficient 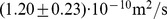 and thus a reduction by a factor of 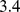 compared to free diffusion in aqueous solution.

## Introduction

Many sensory stimuli are detected at the cilia of sensory cells. Odorant ligands, for instance, are transduced into generator currents in the cilia of olfactory sensory cells, which may serve as a prototype of sensory cilia. As some of the underlying processes are diffusion-limited, any quantitative transduction model would demand, among others, knowledge about to what extent diffusion is slowed down in olfactory cilia. It is generally known that diffusion in neuronal dendrites is non-isotropic and slower than in somata [1]. The size of olfactory cilia, having diameters at least five times thinner than dendrites (200–250 nm [2], [3]), together with the multitude of structural and functional ciliary proteins suggest a further reduction of diffusion in olfactory cilia. Measurements of the diffusion coefficient 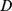 in spermatozoa [4] and in cilia of retina sensory cells [5] are in line with this suggestion but show at the same time that diffusion varies among different types of cilia.

Commonly the diffusion coefficient 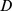 of a certain molecule species would by measured either by observing the fluorescence recovery after photobleaching (FRAP) or by fluorescence correlation spectroscopy (FCS) [6]. However, in the case of tiny, slightly curved and long compartments such as cilia both methods have their limitations. They require either a stable, isotropic FRAP volume without boundaries, or, in the case of FCS, the precise knowledge of the geometry of all diffusional barriers in order to apply the appropriate model with correct parameters [7]. As a consequence, standard FRAP or FCS do not allow to measure diffusion coefficients 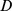 in cilia, and, accordingly, there are almost no published values for 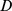 in sensory cilia. The only report on 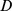 in olfactory cilia is indirect in that it uses cAMP as test molecule and cAMP-gated currents as read-out [8]. This evidence has however been disputed because cAMP is degraded while diffusing [9], and this reaction may vary under different experimental conditions, thereby affecting 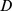. To avoid the latter issue we used an inert fluorophore of similar molecular weight (fluorescein, 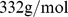; cAMP, 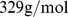) in our study.

Regarding the necessary refinement of FRAP measurements in cilia, Monte Carlo simulations for diffusion in mitochondria and endoplasmatic reticulum suggested that it would be “useful to measure the time course of fluorescence after photobleaching not only in the bleach volume, but at one or more points away from the bleach volume” [10].

We here present a modified FRAP approach along these lines using a confocal, fast scanning, line-illumination microscope [11], where we first bleach fluorescein in one half of a cilium and then observe the fluorophores’ redistribution in the whole cilium. A diffusion model describing the spatiotemporal ciliar fluorescence evolution under such conditions is then used to obtain the diffusion coefficient, with no need to know the diffusional barriers perpendicular to the cilium’s axis.

## Results and Discussion

**Figure 1:**
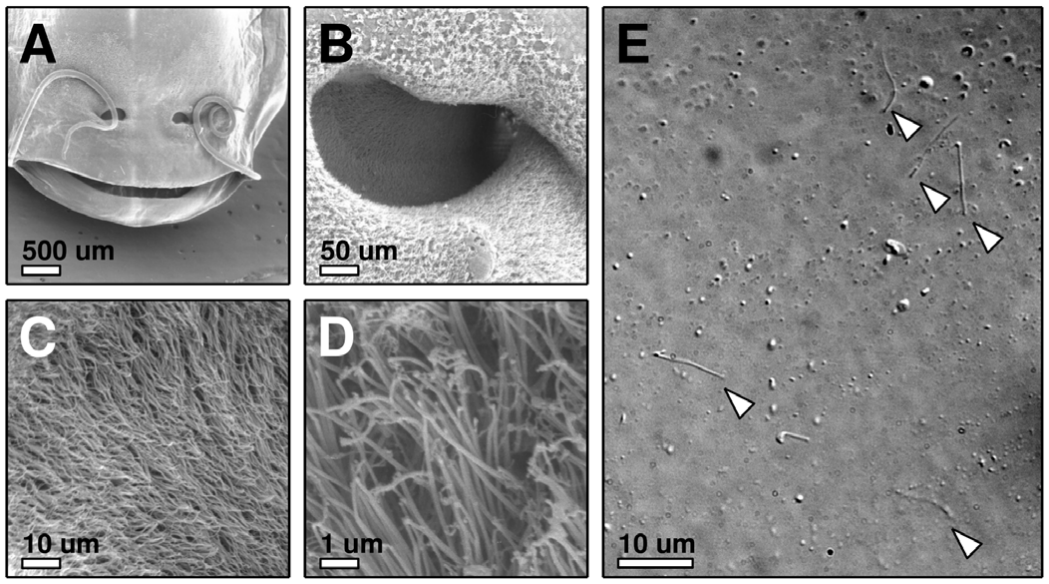
Preparation of olfactory cilia. (A–D) SEM micrographs, (A) Top view onto the nostrils of a Xenopus laevis tadpole, (B) one nostril, at the bottom of which a lawn of sensory clila can be seen (C, D), (E) detached cilia (arrow heads) on a Poly-L-Lysine-coated coverslip, imaged with a 100× objective using DIC.

To study fluorescence dynamics in olfactory cilia we stained the cilia with fluorescein using its non-fluorescent membrane-permeable form fluorescein diacetate, which is cleaved after uptake by intracellular esterases to yield fluorescein. The cilia were detached from the cells by a modified 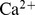 - shock protocol (cf. Methods) and plated on a poly-L-Lysine-coated coverslip (Fig. 1). Under control conditions the fluorescence intensities within a cilium, mapped onto one dimension, were largely constant over the pixels except for some constant deviations owing to pixel-specific offsets of the CCD chip.

**Figure 2:**
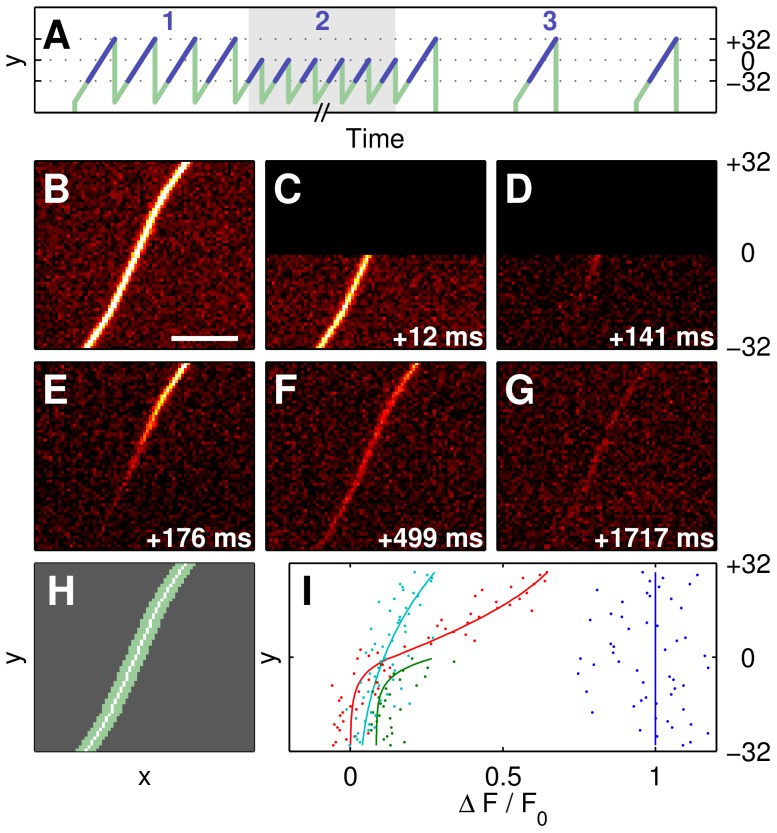
FRAP scanning protocol and sample data. (A) Schematic of the three-phase scanning protocol showing the position of the illumination line in pixel coordinates. In the first phase, several full frames are acquired to determine initial fluorescence. Half-frames are acquired in the second phase at high frame rate (488 f/s) for photobleaching in the lower half of the cilium. The third phase records the fluorescence redistribution due to diffusion at a low frame rate (28 f/s). Image acquisition (blue) is delayed in respect to the mirror position signal (green) for mirror response linearity. Time axis is not to scale, number of images reduced for simplicity. (B–H) Sample frames from all FRAP phases show evolution of fluorescence distribution, scale bar 5 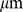, frame times relative to first frame. (B) Initial fluorescence. (C,D) First and last half-frame of the bleaching phase, upper half not imaged and displayed as black. (E) First full frame of the recovery phase shows inhomogeneous fluorescence distribution. (F) Mostly homogeneous distribution after 9 frames in the recovery phase. (G) Last frame of the recovery phase. (H) 2D pixel mask used for maximum projection of 2D intensities onto 1D position on cilium. (I) Projected intensity plots (dots) for selected frames (blue: data from frame B, green: bleaching phase (t = 51 ms), red: E, cyan: F), and corresponding best-fits (solid lines, for full data see Fig. 3B).

We investigated the diffusion within olfactory cilia using a confocal line-illumination microscope [11], where a diffraction-limited line (parallel to the x-direction) was deflected in y-direction, using a three-phase FRAP scanning protocol (Fig. 2A). In phase 1, four images were taken at 325 frames per second to determine the intraciliar fluorescence at the start of the experiment (control, Fig. 2B). The cilium shown was covered by 64 lines, each line containing 512 pixels in x-direction. In phase 2 (bleaching phase), the lower half of the object (32 lines) was scanned at 488 f/s (64 frames, gray area in Fig. 2A), subimages C and D showing examples of frames at the beginning and the end of the bleaching phase. As the upper half of the cilium (black) was not illuminated in phase 2, it was neither bleached nor recorded. In phase 3 (recovery phase) the relaxation of the fluorescence in the cilium was imaged at a low frame rate (28 f/s, subimages E–G).

The confocal 2D data of the cilium were subsequently reduced to one dimension by using templates individually generated from maximum pixel intensities as shown in Fig. 2H. The resulting normalized intensities (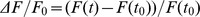 with 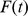 being the masked raw fluorescence intensity at time 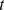, maximum-projected in x-direction, and 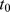 the initial frame time) are shown in Fig. 2I, where the initial intensities (blue) are distributed homogeneously over y. The green curve corresponds to an incompletely bleached cilium (lower part, range [−30,0]). This distribution shows increasingly higher intensities towards the center (range [−10,0]) due to dye molecules diffusing from the unbleached half into the lower half of the cilium, resulting in a partial replenishment. The red and cyan curves represent the fluorescence intensities along the cilium at the beginning and in the middle of the recovery phase, respectively, where the dye molecules diffuse from the upper half into the lower one, while slowly being bleached. Similar data were acquired from 35 cilia.

**Figure 3:**
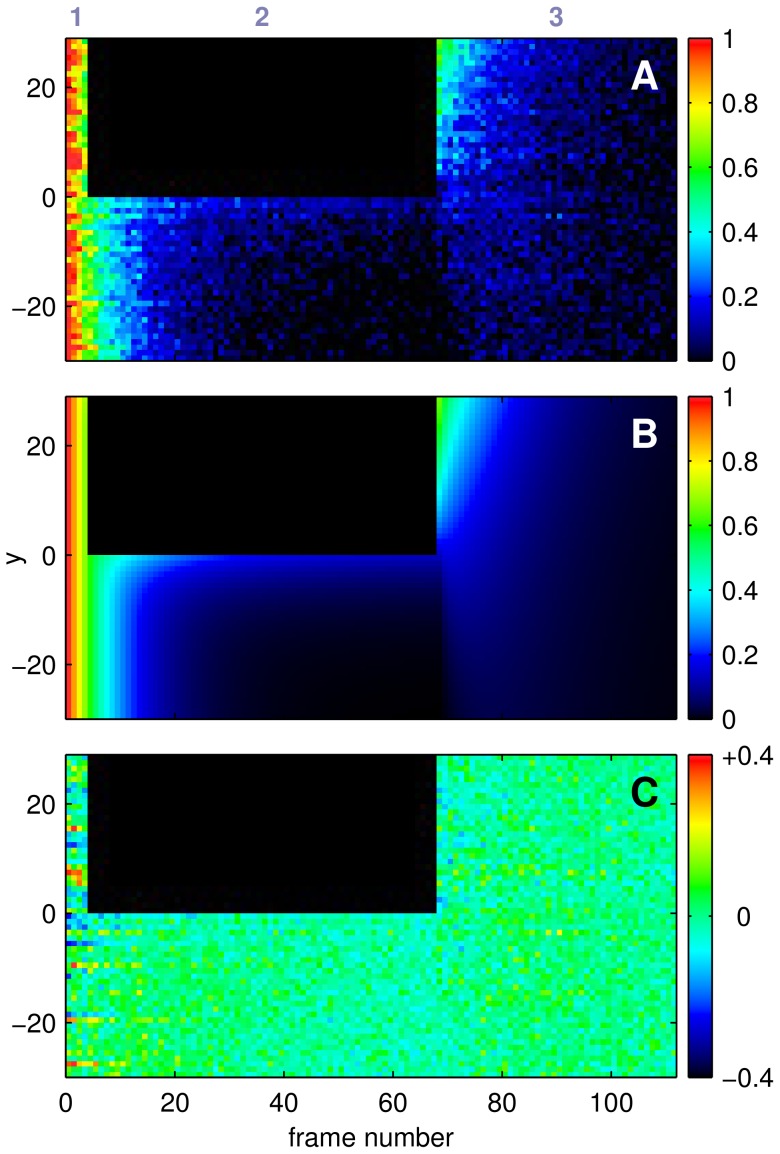
Fit of 1D diffusion model to experimental data. (A) Experimental data, shown as 1D fluorescence distribution over frame numbers for a full experiment, with normalized fluorescence (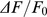) color-coded according to color map. FRAP phases (see Fig. 2A) indicated above. (B) Corresponding data from the best-fit result of the 1D diffusion model. (C) Residuals between A and B, using a smaller colormap range.

Every FRAP measurement of a cilium gives a time-stack of raw data images. This was conveniently reduced, without any loss of information, by (i) mapping the intensities along a cilium onto a line, (ii) color-coding the intensity values, and (iii) repeating the procedure for every raw image. In the resulting representation of the FRAP process (Fig. 3A) every vertical line thus corresponds to the intensity profile along the cilium at the time at which the cilium was scanned. The leftmost vertical stripe (1) represents the control phase consisting of four lines, the adjacent block (2) containing the black rectangle shows the bleaching phase, and the rightmost field (3) corresponds to the recovery phase. The abscissa gives the frame index. Note that the images in the three FRAP phases were taken at different frame rates. The superposition of bleaching and replenishment with dye molecules is best seen at the frontier between bleached and unbleached half.

As the last step of our analysis the diffusion coefficient 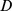 was obtained by modeling the intraciliar fluorescence dynamics prior to, during and after bleaching. Fitting the model to the data then yields the diffusion coefficient 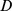. The numerical simulation consists of alternating bleaching and diffusion steps, taking into account the pixel distances 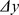, corrected by the angle of the cilium respective to the illumination line (cilia with angles above 45 degrees and overlapping cilia were excluded from the evaluation), and the time delay 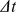 between two frames according to the FRAP phase. For the diffusion simulation we used the one-dimensional, discrete Backward Euler formulation of the diffusion law, i.e.,
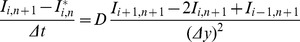
(1)where time (t) and space (y) are discretized as 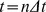, 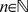, and 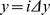, 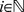. The asterisk (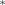) denotes intensities after the photobleaching step (as described below). As the cilia are detached and closed (impermeable) at their ends, we assumed the spatial derivatives at the two boundaries to vanish. With 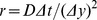, the above system of difference equations can be written as a transform of 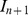 to 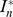,
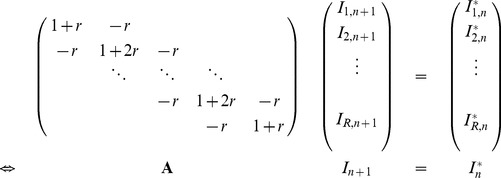
(2)with A being a tridiagonal matrix, which is readily solved by the Thomas method [12]. For the photobleaching steps, the intensities of the illuminated areas 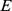 were multiplied with a constant inter-frame photobleaching factor 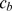, corresponding to a mono-exponential decay.
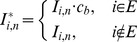
(3)


This is sufficiently accurate for fluorescein at concentrations lower than that of oxygen [13], a condition usually met at room temperature, where we have 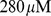 oxygen as compared to approximately 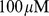 fluorescein.

**Figure 4:**
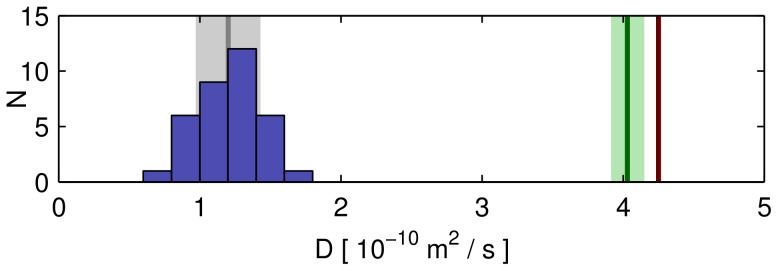
Distribution of diffusion coefficients. Blue: histogram of best-fit results for diffusion coefficients of fluorescein from 35 cilia, with mean and standard deviation as dark gray line and light gray area, respectively. The diffusion coefficient of fluorescein in aqueous solution at 25°C [14] is shown in red, while values corrected for a range of (23±1)°C are shown in green.

The simulated fluorescence distribution 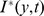 for the complete FRAP experiment was then fitted to the normalized experimental data, using fit parameters 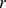, photobleaching constant 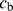, and a fluorescence offset 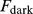 to account for the CCD dark current. The best fit for the experimental data shown in Fig. 3A is given in Fig. 3B, with part C of the figure showing rather homogeneously distributed residues. The resulting fit parameter 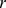 yielded 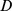 (as 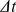 and 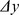 are known). The values for 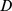 of fluorescein measured in 35 cilia are shown in the histogram of Fig. 4, the average being 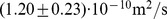.

The variance of 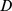 may be explained by different developmental stages and by varying experimental conditions, especially the room temperature. For comparison, the value 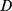 of free diffusion of fluorescein in aqueous solution at 25°C, 
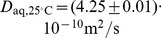
 [14] was corrected to the room temperature of our experiments (23±1)°C using [14].
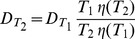
(4)with absolute temperatures 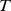 and solution viscosity 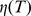. The diffusion in cilia at 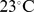 is thus slower by a factor of 3.4 compared to 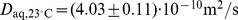.

## Materials and Methods

### Ethics Statement

Tadpoles of *Xenopus laevis* were chilled in a mixture of ice and water and decapitated, as approved by the Göttingen University Committee for Ethics in Animal Experimentation (reference number T24.07).

### Preparation of Olfactory Cilia

Tadpoles of *Xenopus laevis* (stage 52–54; staged after [15]) were chilled in groups of five in a mixture of ice and water and decapitated. Blocks of tissue containing the olfactory mucosa were cut out and incubated for 40 minutes in 1 ml of frog Ringer solution containing (in mM): 98 NaCl, 2 KCl, 1 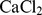, 2 MgCl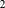, 5 glucose, 5 sodium pyruvate, 10 HEPES, 0.01 fluorescein diacetate and 0.1 MK-571 at pH 7.8. To detach the olfactory cilia, a calcium shock protocol (modified from [16] and [17]) was applied. First, the Ringer solution was exchanged with 0.96 ml of a solution (“solution A”) containing (in mM): 30 TRIS, 100 NaCl, 2 EDTA at pH 8. Then, the calcium concentration was increased by adding 40 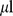 of solution containing 1M CaCl. The solution was kept for 20 minutes at 4 degrees celsius. Detached cilia were separated from the tissue blocks by centrifugation at 3000 rpm (BIOFUGE fresco, Heraeus, Buckinghamshire, England) (sediment discarded) and concentrated by centrifugation at 13000 rpm (supernatant discarded). The sediment was resuspended in solution A and plated onto a microscope slide coated with Poly-L-Lysine.

### Confocal Line Illumination Microscopy

The plated cilia were imaged using a 100× water immersion objective (Achroplan 100×/1,0W, Zeiss, Göttingen) and an upright microscope (Axioskop 2 FS plus, Zeiss, Göttingen) to which a custom-built confocal line illumination unit was attached ([11]). The resulting pixel size was 220 nm. Every cilium was centered in the bright field mode and several confocal images (512×128 pixels) were acquired to determine its total length, position, and orientation. In the automated FRAP experiments we then acquired stacks of full-frame images (512×64 pixels) and half-frame images (512×32 pixels).

### Scanning Electron Microscopy

Specimens were fixed with 1,5% glutaraldehyde and 1,5% paraformaldehyde in 0.1 M sodium phosphate buffer, pH 7.3 for 3 h at room temperature and postfixed for two hours in 2% osmium tetroxide in 0.1 sodium phosphate buffer. After dehydration in graded ethanol, samples for Scanning electron microscopy (SEM) were dried in a critical-point dryer (Polaron, Watford, UK), mounted on stubs, and coated with gold-palladium in a cool sputter coater (Fisons Instruments Uckfield, UK). The specimens were examined in a scanning electron microscope DSM 960 (Zeiss Oberkochen, Germany).

### Data Analysis

Analysis of fluorescence data was performed using custom software written in MATLAB (The MathWorks, Natick, MA), with the time-critical component of diffusion simulation written in C (adapted from [12]).
